# Whole-exome Sequencing of Nigerian Prostate Tumors from the Prostate Cancer Transatlantic Consortium (CaPTC) Reveals DNA Repair Genes Associated with African Ancestry

**DOI:** 10.1158/2767-9764.CRC-22-0136

**Published:** 2022-09-16

**Authors:** Jason A. White, Ernest T. Kaninjing, Kayode A. Adeniji, Paul Jibrin, John O. Obafunwa, Chidiebere N. Ogo, Faruk Mohammed, Ademola Popoola, Omolara A. Fatiregun, Olabode P. Oluwole, Balasubramanyam Karanam, Isra Elhussin, Stefan Ambs, Wei Tang, Melissa Davis, Paz Polak, Moray J. Campbell, Kathryn R. Brignole, Solomon O. Rotimi, Windy Dean-Colomb, Folake T. Odedina, Damali N. Martin, Clayton Yates

**Affiliations:** 1 Tuskegee University, Center for Cancer Research, Tuskegee, Alabama.; 2 Georgia College & State University, Milledgeville, Georgia.; 3 University of Ilorin Teaching Hospital, Nigeria, Ilorin.; 4 National Hospital Abuja, Nigeria, Abuja.; 5 Lagos State University Teaching Hospital, Ikeja, Lagos, Nigeria.; 6 Federal Medical Centre, Abeokuta, Nigeria.; 7 Ahmadu Bello University, Zaria Nigeria.; 8 University of Abuja, FCT, Nigeria.; 9 Molecular Epidemiology Section, Laboratory of Human Carcinogenesis, Center for Cancer Research, NCI, Bethesda, Maryland.; 10 Department of Surgery, New York Presbyterian – Weill Cornell Medicine, New York, New York.; 11 C2i Genomics, New York, New York.; 12 Division of Pharmaceutics and Pharmacology, College of Pharmacy, The Ohio State University, Columbus, Ohio.; 13 University of North Carolina Chapel Hill, North Carolina.; 14 Department of Biochemistry, Covenant University, Ota, Nigeria.; 15 Piedmont Medical Oncology – Newnan, Newnan, Georgia.; 16 Center for Health Equity and Community Engagement Research, Mayo Clinic, Jacksonville, Florida.; 17 Division of Cancer Control and Population Sciences, NCI, Rockville, Maryland.

## Abstract

In this study, we used whole-exome sequencing of a cohort of 45 advanced-stage, treatment-naïve Nigerian (NG) primary prostate cancer tumors and 11 unmatched nontumor tissues to compare genomic mutations with African American (AA) and European American (EA) The Cancer Genome Atlas (TCGA) prostate cancer. NG samples were collected from six sites in central and southwest Nigeria. After whole-exome sequencing, samples were processed using GATK best practices. *BRCA1* (100%), *BARD1* (45%), *BRCA2* (27%), and *PMS2*(18%) had germline alterations in at least two NG nontumor samples. Across 111 germline variants, the AA cohort reflected a pattern *[BRCA1* (68%), *BARD1* (34%), *BRCA2* (28%), and *PMS2* (16%)] similar to NG samples. Of the most frequently mutated genes, *BRCA1* showed a statistically (*P* ≤ 0.05) higher germline mutation frequency in men of African ancestry (MAA) and increasing variant frequency with increased African ancestry. Disaggregating gene-level mutation frequencies by variants revealed both ancestry-linked and NG-specific germline variant patterns. Driven by rs799917 (T>C), *BRCA1* showed an increasing mutation frequency as African ancestry increased. BRCA2_rs11571831 was present only in MAA, and BRCA2_rs766173 was elevated in NG men. A total of 133 somatic variants were present in 26 prostate cancer–associated genes within the NG tumor cohort. *BRCA2* (27%), *APC* (20%), *ATM* (20%), *BRCA1* (13%), *DNAJC6* (13%), *EGFR* (13%), *MAD1L1* (13%), *MLH1* (11%), and *PMS2* (11%) showed mutation frequencies >10%. Compared with TCGA cohorts, NG tumors showed statistically significant elevated frequencies of *BRCA2*, *APC*, and *BRCA1*. The NG cohort variant pattern shared similarities (cosign similarities ≥0.734) with Catalogue of Somatic Mutations in Cancer signatures 5 and 6, and mutated genes showed significant (*q* < 0.001) gene ontology (GO) and functional enrichment in mismatch repair and non-homologous repair deficiency pathways. Here, we showed that mutations in DNA damage response genes were higher in NG prostate cancer samples and that a portion of those mutations correlate with African ancestry. Moreover, we identified variants of unknown significance that may contribute to population-specific routes of tumorigenesis and treatment. These results present the most comprehensive characterization of the NG prostate cancer exome to date and highlight the need to increase diversity of study populations.

**Significance:**

MAA have higher rates of prostate cancer incidence and mortality, however, are severely underrepresented in genomic studies. This is the first study utilizing whole-exome sequencing in NG men to identify West African ancestry-linked variant patterns that impact DNA damage repair pathways.

## Introduction

For men, adenocarcinoma of the prostate is the most frequently diagnosed cancer, accounting, globally, for an estimated 1,414,259 cases and 375,304 deaths in 2020. The preponderance of this mortality is for men of African ancestry (MAA), including African American (AA), Central American, Caribbean, and Sub-Saharan African men (1). The Globocan 2020-derived mortality-to-incidence ratio, “an indirect description of the general survival experience” of prostate cancer in Africa, is 0.55, relative to 0.32 and 0.17 for Asia and Europe, respectively (2, 3). Furthermore, studies of AA men showed higher incidence, worse prognoses, and higher mortality compared with European American (EA) men (4, 5). Although there is a substantial contribution of social and environmental influence on the disparity, emerging evidence from genomic profiles suggests that this disease is highly heterogenous (6), and its etiology and phenotype are influenced by enrichment of African ancestral genetic markers, with West African ancestry linked with higher Gleason grade at diagnosis (7–9).

The racial disparity in prostate cancer biology is typically characterized by increased genomic mutations, resulting in a more aggressive phenotype. For instance, West African ancestry is associated with distinctive somatic genomic mutations (10, 11). Conversely, understanding these putatively targetable genomic mutations presents opportunities for effective population-relevant and genomics-guided interventions that can improve clinical outcomes. This relies on the availability of genomic data for tumors from MAA. However, despite an upsurge in genomic data for human cancers, the data on prostate cancer from African sources are grossly underrepresented in the literature and genomics databases. For instance, AA samples account for only about 10% of The Cancer Genome Atlas (TCGA) prostate cancer sample cohorts (11, 12). Consequently, this gross underrepresentation impedes deciphering of clinically actionable genomic mutations that could be used to develop precision interventions for MAA. Hence, it is imperative to increase the representation by sequencing the tumor genome of prostate cancer in MAA.

Although Black men in the Americas generally have ancestral roots in the Atlantic coasts of Africa (13–15), the translational impact of genomics data of AA men to indigenous Africans is limited because of the varying proportions of European-related and intra-African admixtures of African American (16, 17). Such admixture and variation in germline mutations influence gene expression and phenotype (18); and are limiting factors in the understanding of the contribution of genetics to health disparities (19). Hence, studying the genomic architecture of prostate cancer in the indigenous African population is essential for advancing understanding of the contribution of African genetics to prostate cancer biology and the phenotype of this disease in the African Diaspora. To date, inadequate attention has been given to generation of genomics studies of prostate cancer among indigenous Africans. Aside from three genome-wide association studies of prostate cancer in Ghanaian (20), Ugandan (21), and South African (22) men, only Jaratlerdsiri and colleagues (23) have reported whole-genome sequence data on tumors from indigenous sub-Saharan Africans. Their analysis of prostate cancer of six South African Black men identified distinctive and elevated oncogenic driver mutations, with a high proportion of these recurrent mutations appearing early in tumorigenesis. They also showed that tumors of the African men they studied had fewer complex genomic rearrangements, loss of *PTEN*, and absent *ERG* fusions and *PIK3CA* mutations relative to AAs. Furthermore, apart from large deletions within the *BRCA2*, *DEFA1B*, and *MFF* genes, they did not report any pathogenic mutations in high-penetrance genes, such as *BRCA1*, *BRCA2*, *ATM*, and *CHK2* among the South African cohort (23, 24). Previous studies have identified, for prostate cancer of AAs, a high burden of mutations in these DNA repair genes (25, 26); suggesting that PARP inhibitors could improve clinical outcomes for men of African ancestry with prostate cancer (27). The South African study is limited by the small sample size. Furthermore, the differences observed could be due to the low contribution of South-African Khoe-San ancient ancestry genes to the AA genetic pool, which is a source of bias (16, 17). It is therefore our hypothesis that genomic profiling of prostate cancer in indigenous West African men will identify clinically actionable targets for precision intervention for MAA. The utility of clinical mutational profiling necessitates greater emphasis on identifying variants that can be used to help these underrepresented patients.

The aim of the current study was to analyze whole-exome sequencing (WES) of 45 Nigerian (NG) primary treatment-naïve formalin-fixed, paraffin-embedded (FFPE) prostate cancer and 11 NG nontumor prostate samples collected within the Prostate Cancer Transatlantic Consortium (CaPTC). Study of prostate cancer of NG men allowed us to provide genetic information of the indigenous West Africa population with the highest genetic contribution to AA men (16) and provide opportunities to investigate the shared genetic background of both groups for causal disease variants. As such, our data will be relevant for deriving actionable clinical information for prostate cancer intervention for MAA.

## Materials and Methods

### Sample Collection and Genomic DNA Extraction

**FIGURE 1:**
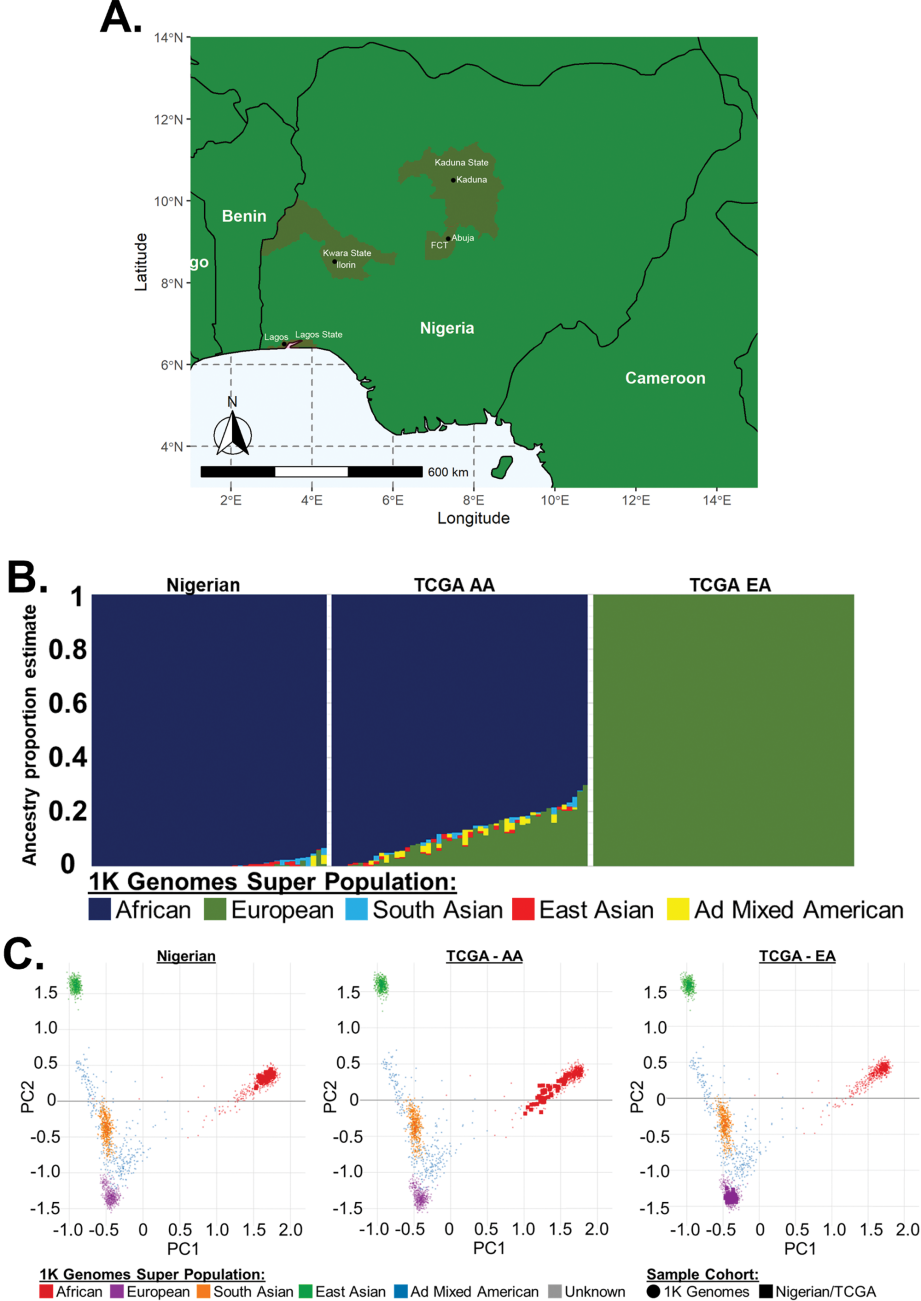
Sample collection sites and genetic admixture analysis. **A,** At clinical sites across Nigeria, 45 samples were collected. **B,** Admixture v1.3.0 was used to estimate ancestry proportions, based on reference populations from the 1000 Genomes Project phase III superpopulations. Prior to analysis, rare variants (i.e., <5% across all phase III 1000 genomes), all indels, and any SNPs that were not biallelic were removed. Samples within the CaPTC cohort had an average African proportion of 99.1%. TCGA Samples (*n* = 50) with >70% African ancestry were classified as AA; 402 TCGA samples contained >60% European admixture. Those samples were sorted by European proportion, and the top 50 samples were classified as EA and utilized in this study. The average European proportion of this group was 99.996%. **C,** Germline variants within the NG and TCGA cohorts were compared with phase III 1000 Genomes superpopulations using principal component analysis. NG samples strongly clustered with the African superpopulation. AAs samples clustered with the African superpopulation; European samples clustered with the European superpopulation.

This study utilized 45 FFPE advanced-stage, treatment-naïve primary prostate cancer and 11 NG nontumor prostate samples collected from four participating clinical sites within the CaPTC network (Fig. 1A). In accordance with the U.S. Common Rule, the archived samples used in this study were reviewed and approved by the Institutional Review Boards of their respective clinical institutions (University of Ilorin Teaching Hospital, National Hospital Abuja, Lagos State University Teaching Hospital, Federal Medical Centre, Ahmadu Bello University, and the University of Abuja) and by the Institutional Review Board at Tuskegee University (Tuskegee, AL). Because of the retrospective nature of this study and the usage of deidentified archived samples, the review boards deemed informed written consent to be unnecessary for this study. Five 10-μm-thick curls were obtained from each block with >50% tumor and ≤50% necrosis and shipped to Q2 Solutions for DNA extraction and quality analysis. Following the manufacturer's protocol, genomic DNA and total RNA were purified using Allprep DNA/RNA FFPE kits (Qiagen). DNA quality and quantity were checked with Qubit 2.0 fluorometry (Life Technologies) and with KAPA hgDNA quantification and QC kits [Kappa Biosystems (Roche)]. DNA quality and quantity thresholds were >0.2 μg and a Q129/Q41 ratio >0.00225, respectively.

### Pathologic Scoring

The FFPE blocks were processed at the Pathology Biorepository Shared Service (PBSS) core at the University of Maryland, Baltimore (Baltimore, MD). For pathology review, an initial hematoxylin and eosin (H&E) slide was prepared from each block. A pathologist assessed the presence and quantity of tumor, and the presence and quantity of normal tissue. Up to two tumor and normal cores per H&E slide were circled, and the total number of cores per slide was recorded in a sample manifest. In addition, the pathologist provided the tumor core(s) Gleason score for each tumor circle. Those FFPE blocks with sufficient tumor and/or normal cores available were submitted to the PBSS research histology lab for core extraction. Using the corresponding H&E slide as a template, cores were extracted from each FFPE block by use of a manual tissue core extraction device with RNAse/DNAse-free conditions. The cores were placed in labeled RNAse/DNAse-free cryovials. A sample manifest for the extracted cores accompanied samples shipped to the NIH Laboratory of Human Carcinogenesis.

### WES

As outlined in Supplementary Fig. S1, library preparation was performed using Agilent SureSelectXT Human All Exon V6 r2 Exome Kits (Agilent Technologies). Sequencing (2 × 150 bp) was performed on either an Illumina HiSeq4000 or on an Illumina NextSeq500 (Illumina), to a target of 100 (± 10) million raw data reads per each sample library. Following sequencing, raw fastq files were transferred to the NIH Biowulf supercomputing cluster and analyzed using the Center for Cancer Research Collaborative Bioinformatics Resource (CCBR) whole-exome pipeline (https://github.com/CCBR/Pipeliner). Reads were trimmed using Trimmomatic v0.33 (28) and mapped to the hs37d5 version of the human reference genome (ftp://ftp.1000genomes.ebi.ac.uk/vol1/ftp/technical/reference/phase2_reference_assembly_sequence/hs37d5.fa.gz) using BWA-MEM v07.17 (29). Binary Alignment Map (BAM) files were processed using Samtools v1.8 (http://www.htslib.org/; ref. 30), and Picard v1 (http://broadinstitute.github.io/picard/) was used to mark duplicates. GATK v3.8 (31) was used to perform indel realignment and base recalibration. Read and alignment-level quality analysis was performed using Qualimap v2.2.1 (32). Alignment quality metrics were analyzed and visualized using RStudio 1.2.5003 (http://www.rstudio.com) [R 3.6.3 (https://www.R-project.org)], the ggstatsplot v0.3.0 (https://indrajeetpatil.github.io/ggstatsplot/) package, and the compareGroups v4.2.0 (33) package.

### Variant Calling and Filtration

Germline variant calling was completed using HaplotypeCaller (34), and Annovar v2019Oct24 was used for variant annotation. Variant filtration followed GATK Best practices. Cohort SNPs and indels were separated, filtered, and recombined for downstream analysis. SNP filters were Qual < 30.0, QD < 2.0, FS > 60.0, MQ < 40.0, MQRanksum < −12.5, and ReadPosRankSum < −8.0. Indel filters were Qual < 30.0, QD < 2.0, FS >200.0, and ReadPosRankSum < −20.0. Once the variants were recombined, cohort germline variants were compared with a ClinVar-derived list of known prostate cancer–associated germline variant regions (Supplementary Table S1) to target variants of known clinical importance. Germline variants with a read depth >3 and a variant allele frequency >50% were retained for downstream analysis (Supplementary Table S2). Somatic variant calling was completed using MuTect2 (35), and Annovar v2019Oct24 (36) was used for variant annotation. As described by Jones and colleagues, a single unmatched NG normal FFPE exome, sequenced using the same methods as for the tumor samples, was paired with each tumor exome to filter false-positive variant calls (37). Mutation tables were imported into RStudio for visualization and analysis using the maftools (ref. 38; v2.4.10) package. Variants were (i) screened for strand bias (Supplementary Fig. S2) using GATK FisherStrand Phred Score, (ii) separated into variants within known prostate cancer–associated genes and variants within novel prostate cancer–associated genes (Supplementary Table S3), using ClinVar and (iii) and filtered (Supplementary Fig. S3) using two filtering regimes. After filtering, retained variants within genes identified in ClinVar were considered “Known”; conversely, variants within genes not identified in ClinVar were called “Novel.” Filtering steps included (i) exclusion of silent and non–protein-coding mutations, (ii) variant allele read depth ≥3, (iii) variant allele frequency >5% (10% for variants within novel prostate cancer–associated genes), (iv) dbNSFP (39) Genome Aggregation Database (gnomAD; ref. 40) exome allele frequency <0.01 (<0.001 for variants within novel prostate cancer–associated genes and variants lacking allele frequencies were retained for downstream filtration), (v) identification as Pathogenic or Uncertain in ClinVar v 20200419 (41), (vi) removal of dbSNP-annotated variants identified in NG unmatched normal samples (*n* = 11; Supplementary Table S4), (vii) present in genes altered in least 5% of tumors, and (viii) manual validation in the Integrative Genomics Viewer (IGV; Supplementary Fig. S4). For genes mutated across at least five prostate cancer samples, Fishers exact test was used to compare cohort mutation frequencies. The test was completed using the maftools clinicalenrichment function. *P* values < 0.05 were considered significant.

### TCGA PRAD Data Acquisition and Analysis

Access to TCGA Prostate adenocarcinoma (PRAD) data (Accession: phs000178.v11.p8) was obtained through the database of Genotypes and Phenotypes (dbGAP). Raw sequencing files in BAM format were downloaded through the Genomic Data Commons (GDC) data transfer tool from the GDC Data Portal (https://portal.gdc.cancer.gov/). After download, the raw files were sorted using Samtools and split into their constituent forward and reverse fastq files using bedtools v2.29 (42). Once separated, the fastq files were processed through the CCBR whole-exome pipeline and analyzed using the same methods as for NG CaPTC samples.

### Genetic Admixture Estimation

To ensure accurate ancestral group assignment, HaplotypeCaller (43) and Admixture v1.3.0 (44) were used to estimate ancestry proportions, based on reference populations from the 1000 Genomes Project phase III superpopulations in all TCGA and NG samples (Supplementary Fig. S5). Rare variants [i.e., <5% across all phase III (45) 1000 genomes], all indels, and any SNPs that were not biallelic were removed prior to analysis. TCGA samples (*n* = 57) with majority African ancestry were classified as AAs. AAs are highly admixed, which in turn increases genetic variation (17, 46). Because our NG population has greater than 90% African Ancestry, we focused on AAs within TCGA that have at least 70% African admixture (*n* = 50) as a comparison group, which has been used in previous reports. Thus, the AA patients with TCGA that have African admixture at or above 70%, represent 87% of the total AA samples in the prostate cancer TCGA. The EA samples that at or above >60% European admixture represent 402 TCGA samples. Those samples were sorted by European proportion, and the top 50 samples were classified as EAs and utilized in this study. The average European proportion of this group was 99.996%.

### COSMIC Signature Enrichment

Using the maftools package, filtered single-nucleotide variants across each cohort were used to estimate the representation of Catalogue of Somatic Mutations in Cancer (COSMIC; cancer.sanger.ac.uk; ref. 47) mutation signatures within each tumor sample. Maftools uses cophenetic correlation and nonnegative matrix factorization to determine the optimal number of SNP signatures (across the cohort), extracts those signatures, and compares them with the known (*n* = 30) COSMIC signatures.

### Variant Functional Gene Ontology and Network Analyses

Filtered variants, present in at least two NG prostate cancer tumor samples, were imported into Cytoscape (48) (v. 3.7.2) to assess functional gene ontology enrichment and to visualize the GO term interaction network. Once separated, functional analysis and network construction were completed using the stringApp (ref. 49; v 1.6.0) and ClueGO (ref. 50; v. 2.5.7) plug-ins. A two-sided (enrichment/depletion) hypergeometric test with Bonferroni step-down was used to determine Reactome Pathways (v. 08.05.2020) enrichment. Analysis thresholds included enrichment significance of *P* ≤ 0.01, a minimum of 5% gene inclusion and a kappa score threshold of ≥0.4. ClueGO uses kappa scores to determine the likelihood of GO term interactions and groupings.

### Data Availability

The data generated in this study are publicly available in dbGaP (Accession phs002547.v1.p1).

## Results

To determine the somatic and germline variants associated with prostate cancer in NG men, we collected samples from multiple institutions within the CaPTC. Specifically, we collected 45 intermediate (Gleason scores 4 + 3) and high-grade (Gleason scores ≥4 + 3) tumors and 11 nontumor prostate samples. Samples averaged >68 × coverage, 57.2 mapping quality and 263 million mapped reads per sample (Supplementary Table S5). A total of 31 (20 tumor and 11 nontumor) samples were collected from Northern Nigeria), 17 samples from Central Nigeria, and eight samples from Southwest Nigeria (Supplementary Table S6). PRAD exome data were downloaded from TCGA, using the dbGAP database as a comparison cohort (Supplementary Table S7).

Race is a poor group classifier for linking genetic variation and disease causation (51); moreover, self-reported race can obscure genetic variation due to misunderstandings about family heritage, cultural influences, and/or other societal factors (52). To ensure that our NG and TCGA cohort comparisons were not skewed by bias within self-reported race, we quantified the individual genetic admixture within each patient sample. To accomplish this, germline SNPs were compared with 1000 genomes superpopulations (African, European, South Asian, East Asian, and admixed American), and ancestry proportion estimates were calculated (Fig. 1B). NG patients showed an average genetic ancestry of 99.1% African. The genetic ancestry of TCGA AA patients was predominantly a mixture of African (50.2%–99.99%) and European admixture (1%–43%). To reduce the impact of this variance on our analysis, we selected only TCGA AA patients with ≥70% African ancestry (*n* = 50). TCGA EA patients showed minimal admixture, with >98.3% European ancestry. Nine patients self-identified as EAs possessed ≤45% European ancestry. Four of the 9 patients were majority (>50%) admixed American, 2 were majority East Asian, 2 were majority African, and 1 was predominantly (45%) European with 35% admixed American and 16% African admixture. To obtain an EA comparison group, we sorted (high to low) the cohort by European ancestry proportion and selected the top 50 TCGA EA patients. After admixture estimation and sample selection, principal component analysis plots were used to visualize the relationships between each cohort and the five 1000 Genomes superpopulations (Fig. 1C). The NG and TCGA EA cohorts clustered with their ancestral 1000 genomes superpopulations, and TCGA AA cohort clustered with the African superpopulation. Thus, data for these patients were used in subsequent analyses.

**FIGURE 2:**
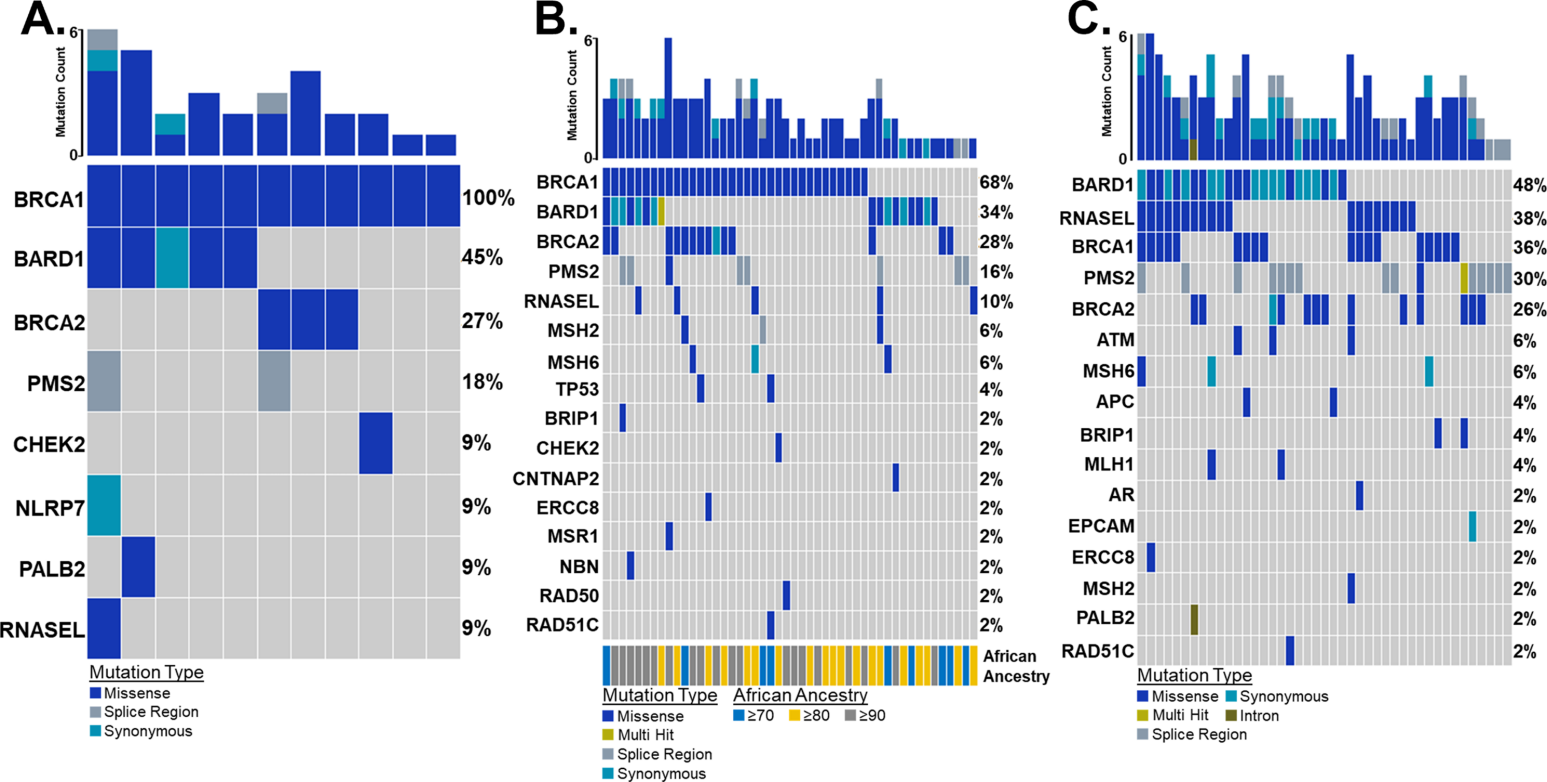
Prostate cancer germline variant oncoplot. **A,** NG germline variants detected across 11 normal samples were filtered against known ClinVar cancer variants. In at least two tumor samples, four genes known to harbor cancer-related variants were mutated. These genes included *BRCA1* (BRCA1 DNA repair associated)—100%, *BARD1*—45%, *BRCA2* (DNA repair associated)—27%, and *PMS2* (PMS1 homolog 2, mismatch repair system component)—18%. As a comparison with NG prostate cancer exome samples, TCGA prostate cancer samples (*n* = 50 AA and *n* = 50 EA) were downloaded through dbGAP and analyzed for genetic variants. **B,** In the AA cohort. eight genes showed germline mutations in at least two samples. **C,** In the EA cohort, 10 genes showed germline mutations in at least two samples.

**FIGURE 3:**
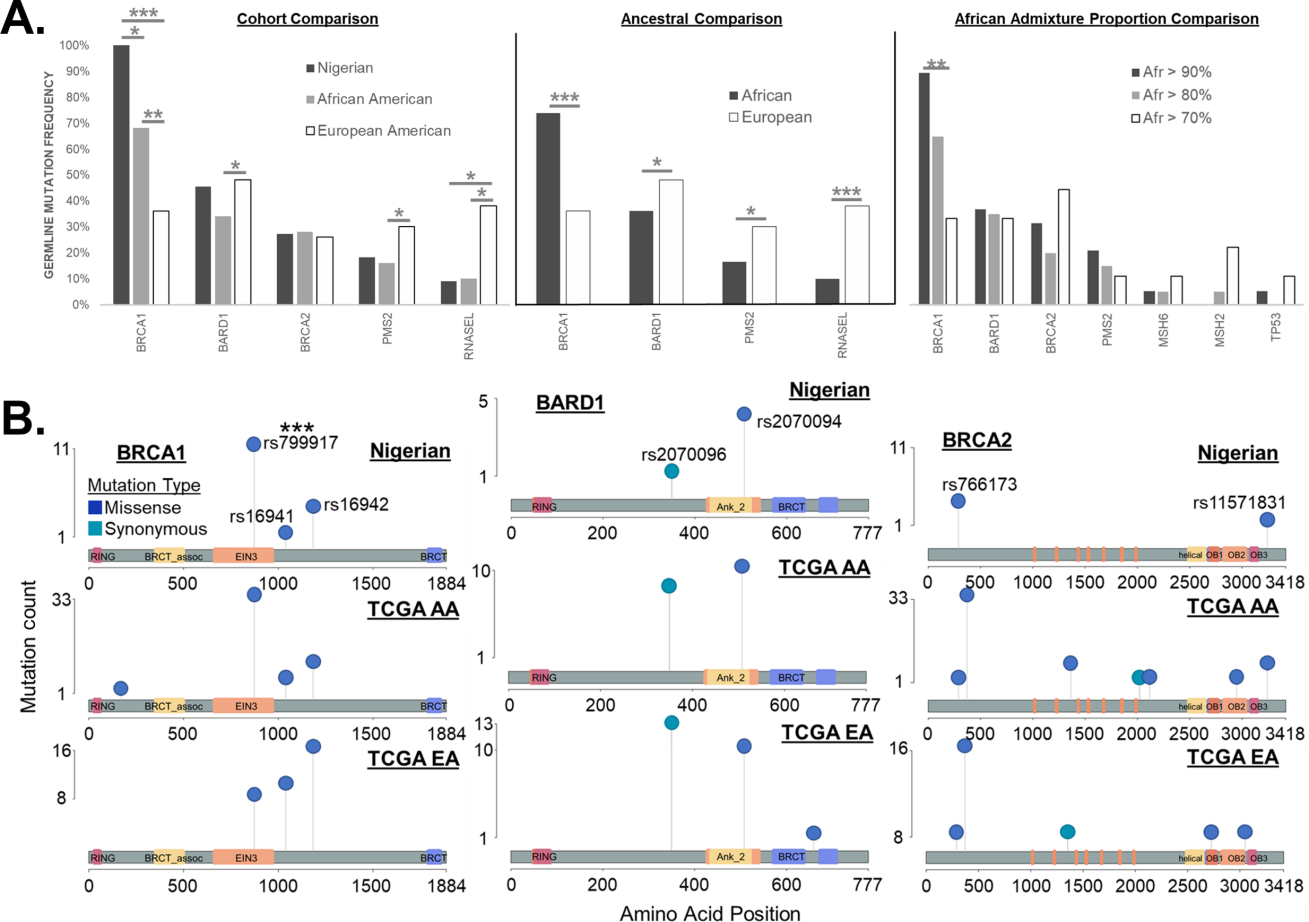
NG prostate cancer cohort germline mutations comparison with TCGA prostate cancer cohorts and lollipop plots. **A,** Prostate cancer of NG and AA men showed more *BRCA1* germline mutations (*P* ≤ 0.001 and *P* ≤ 0.01, respectively) compared with European men. In addition, prostate cancer of NG men showed more (*P* ≤ 0.036) *BRCA1* mutations relative to AA men. In prostate cancer of EA men, *BARD1* was mutated at a higher rate (*P* ≤ 0.048). *BRCA2* showed no significant difference in cohort mutation rates. AA men with greater than 90% African ancestry (*n* = 19) show a statistically significant (*P* ≤ 0.021) increase in *BRCA1* germline mutations when compared with those with lower amounts of African ancestry (*n* = 29). In addition, AA men with 90% African ancestry have a statistically higher (*P* = 0.012) frequency of the *BRCA1* variant rs799917. **B,** To disaggregate mutation rates down to specific variants, lollipop plots revealed a finer variation in cohort patterns. The ancestry-linked pattern of *BRCA1* is driven by rs799917, which was more frequent (*P* ≤ 0.001) for men of African ancestry. rs16941 and rs16942 were elevated in prostate cancer of European men, but that difference was not statistically significant. *BARD1* germline variants showed no significant difference in variant rates; however, the patterns appeared to be specific to NG men. Compared with AA and EA cohorts, rs2070096 was lower and rs2070094 was higher. *BRCA2* germline variants displayed no statistically different variant rates, but both ancestry-linked and NG-specific patterns were discernable. rs11571831 was present only in prostate cancer of men of African ancestry, and rs766173 was elevated in prostate cancer of NG Men. *P* values were produced via two-sided Fisher exact test groupwise comparison.

The NG cohort harbored 31 known, non-benign, germline variants. Four genes [*BRCA1* (100%), *BARD1* (45%), *BRCA2* (27%), and *PMS2* (18%)] were altered in at least two samples (Fig. 2A). These genes also showed top mutation frequencies within both TCGA cohorts (Fig. 2B and C). Across 111 germline variants, the AA TCGA cohort reflected a pattern [*BRCA1* (68%), *BARD1* (34%), *BRCA2* (28%), and *PMS2* (16%)] similar to that for NG samples. In addition, the rate of *BRCA1* mutations increased (*P* ≤ 0.021) as African admixture increased (Fig. 3A; Supplementary Table S8). A total of 126 germline variants were present in the EA TCGA cohort. Disaggregating mutation frequencies down to specific variants revealed both ancestry-linked and NG-specific germline variant patterns. *BRCA1* showed an increasing mutation frequency as African admixture increased (Fig. 3A). That pattern was driven by three variants (rs799917, rs16941, and rs16942; Fig. 3B). The frequency of rs799917 was higher for men of African ancestry; rs16941 and rs16942 were lower. In esophageal squamous cell carcinoma, the BRCA1_rs799917 T>C SNP inhibits mir-638–mediated regulation of *BRCA1*, thus reducing *BRCA1* expression and increasing cancer cell proliferation (53). This variant is also linked to a higher risk of gastric, lung, and triple-negative breast cancer (54–56). BRCA1_rs16941 and BRCA1_rs16942 are variants of unknown significance (VUS). *BARD1* germline variant patterns appear to be specific to NG men. Compared with AA and EA cohorts, rs2070096 is lower and rs2070094 is higher. Of note, the BARD1_rs2070094 SNP resides within the BARD1-binding domain of *BRCA1* and may provide a protective function that enhances DNA repair by enhancing *BARD1/BRCA1* binding stability (57). BARD1_rs2070096 is a VUS. *BRCA2* germline variants displayed both ancestry-linked and NG-specific patterns. rs11571831 was present only in men of African ancestry, and rs766173 was high in NG men. Both *BRCA2* variants are classified as VUS. Although most variants in NG prostate cancer were identified as VUS, their presence and differing frequencies, compared with TCGA, provide opportunities for future investigations. Characterizing the full mutation spectrum is a first step in understanding how best to diagnose and treat this underrepresented patient population.

Somatic variant analysis of tumor-only sequencing data involves multiple nontrivial steps that are distinct from the analysis of matched tumor and normal sequencing. Therefore, we used an established pipeline that incorporated a panel of normal samples. We used an unmatched NG normal sample to filter out NG-specific germline variants (37, 58), reducing the unique NG variants by 70.8% from 2,506,254 to 730,285 variants (Supplementary Fig. S6). Within TCGA cohorts, we used each sample's patient-matched normal, which produced 11,208 unique AA variants and 15,191 unique EA variants. Because the NG cohort contained many somatic variants, we employed two filtering regimes, one for variants within known prostate cancer–associated genes (as identified in ClinVar) and one for variants within genes not associated with prostate cancer. We identified 905 variants across 25 genes known to be associated with prostate cancer, and 156 variants across 51 novel prostate cancer genes. Using the same approach, we identified 15,854 variants in TCGA AA cohort and 21,957 variants in TCGA EA cohort (Supplementary Table S9). Consistent with other sequencing studies (59), our results showed the same racial mutation patterns for *SPOP, ATM, TP53,* and *PIK3CA*. TCGA cohorts did not show recurrent mutations in genes not associated with prostate cancer. Our dual filtering approach allowed us to filter, independently, each set of variants across the NG cohort without overfiltering variants within known prostate cancer–associated genes and to identify high-confidence variants in novel prostate cancer–associated genes.

**FIGURE 4:**
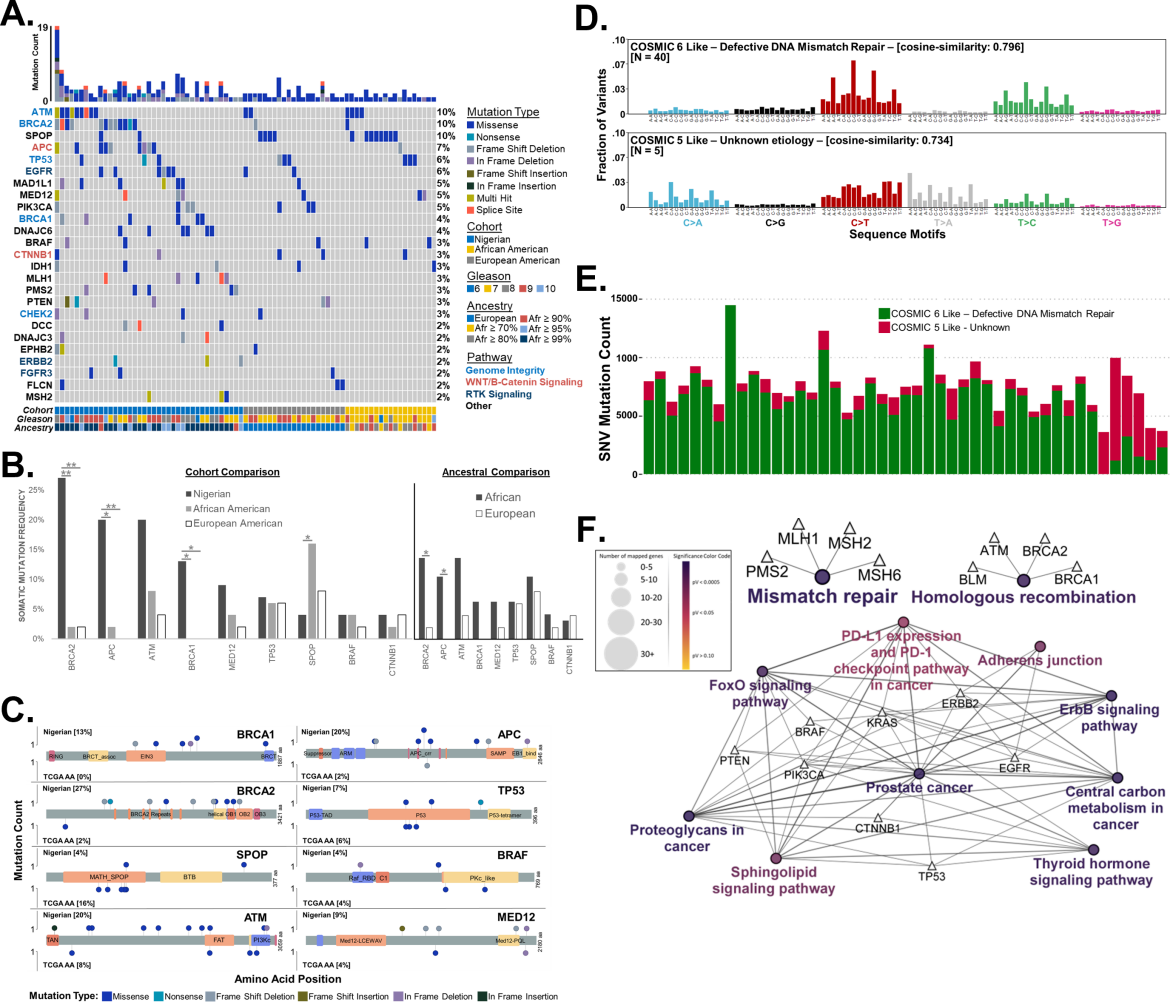
Prostate cancer somatic variants within known prostate cancer–associated genes. **A,** Variant calling within the NG cohort (*n* = 45) produced 1,168,250 variants. A total of 25 genes were known to be associated with prostate cancer harbored variants in at least two tumor samples. The most frequently mutated of these included *BRCA2* (BRCA2 DNA repair associated)—27%, *APC* (APC regulator of WNT signaling pathway)—20%, *ATM* (ATM serine/threonine kinase)—20%, *BRCA1* (BRCA1 DNA repair associated)—13%, and *DNAJC6* [DnaJ heatshock protein family (Hsp40) member C6]—13%. As a comparison with NG prostate cancer exome samples, TCGA prostate cancer samples (*n* = 50 AA and *n* = 50 EA) were downloaded through dbGAP and analyzed for genetic variants. **B,** Prostate cancer of NG men showed a significant (*P* ≤ 0.01) elevation in *BRCA2* somatic mutations compared with African and EA men. A significant increase was also evident for *APC* (*P* ≤ 0.05) and *BRCA1* (*P* ≤ 0.05). Compared with NG men, prostate cancer of AAs were elevated (*P* ≤ 0.05). NG and AA men also showed higher, but not significant, mutation frequencies of *ATM, MED12,* and *BRAF*. **C,** Somatic mutations for NGs and AAs were distributed across the amino acid sequence of the most mutated genes. None of the variants were shared across or within cohorts. **D** and **E,** SNPs in the NG prostate cancer cohort were compared with known cancer-related mutation signatures within the COSMIC. A total of 89% of NG prostate cancer mutation patterns were similar (cosign similarity ≥0.796) with COSMIC signatures 6. The remaining 11% were more like COSMIC 5. **F,** Mutated genes (*n* = 83) present in at least two NG prostate cancer tumor samples (*n* = 45) were imported into Cytoscape to assess functional gene ontology enrichment and visualize the GO term interaction network, using Kyoto Encyclopedia of Genes and Genomes pathways. Variants showed significant (*q* ≤ 0.000538) GO and functional enrichment across multiple GO groups, including mismatch repair, homologous recombination, prostate cancer, and several cancer-related signaling pathways.

Within the NG cohort, 133 somatic variants were present in 26 prostate cancer–associated genes. Nine genes [*BRCA2* (27%), *APC* (20%), *ATM* (20%), *BRCA1* (13%), *DNAJC6* (13%), *EGFR* (13%), *MAD1L1* (13%), *MLH1* (11%), and *PMS2* (11%)] showed mutation frequencies >10% (Fig. 4A). Of NG prostate cancer, 53% showed mutations in genes (*BRCA2, ATM, BRCA1, CHEK2, TP53*, and *MSH6*) associated with genome integrity. TCGA AA and EA cohorts harbored 67 and 73 somatic variants, respectively. Across both cohorts, fifteen genes were mutated in at least two samples (*SPOP, ATM, TP53, BRAF, MED12, PIK3CA, CTNNB1, EGFR, FLCN, MYH7, PTEN,* and *TTN*). *SPOP* and *ATM* were the most frequently mutated genes in AA tumors and were mutated two times more compared with EA. Comparison of the mutation frequencies between TCGA cohorts did not show any statistically significant differences; however, AA tumors showed a significant increase in SPOP mutations compared with NG (Fig. 4B). *BRCA2, APC,* and *BRCA1* showed statistically significant increases in the NG cohort. Though not statistically significant, *ATM* had the highest mutation frequency associated with increasing African ancestry; specifically TCGA EAs had an *ATM* mutation frequency of 4%, but TCGA AA and NG cohorts had rates of 8% and 20%, respectively. Somatic mutations for NGs and AAs were distributed across the amino acid sequence of the most mutated genes. None of the variants were shared across or within cohorts. (Fig. 4C). Comparison of EA and NG cohorts showed no discernible pattern (Supplementary Fig. S7); however, the EA cohort did show a number of variants within a known SPOP hotspot.

In addition to the variants within known prostate cancer driver genes, we identified four novel mutated genes that showed mutation frequencies >10%. *CACNA2D2* had the highest mutation rate of 29% (Supplementary Fig. S8A) and showed a recurrent (*n* = 13) missense SNP of Leu54Phe (rs569543350; Supplementary Fig. S8B). *TTN* (Titin) had the second highest mutation frequency at 20%. The size of this large protein (>30,000 amino acids) renders it more susceptible to DNA repair errors, making the functional significance of these mutations unreliable, even after rigorous variant filtering (60–63). *SYNE1* was the third most frequently mutated gene (16%) in the novel prostate cancer set. This gene showed a recurrent (*n* = 2) missense SNP of Gln1491Glu. The fourth most mutated gene was *ADAMTS2*. This gene showed a recurrent (*n* = 4) in-frame insertion of Leu_Pro29dup and an overall mutation frequency of 13%. Finally, 47 other genes not known to be associated with prostate cancer were mutated in 2 or more patients; however, we did not characterize these due to their low mutation frequencies.

To validate the observed germline variant frequencies, we analyzed NG tumors using the germline variant pipeline (Supplementary Fig. S9). NG tumor samples not only possessed comparable rates of gene-level variation [*BRCA1* (100%), *BARD1* (41%), and *BRCA2* (18%)] but also showed comparable variant frequencies.

We next investigated the overall mutational patterns within each cohort to understand global somatic events. The NG cohort shared similarities (cosign similarities ≥0.734) with COSMIC signatures 5 and 6 (Fig. 4D). Five NG cohort samples had a mutational pattern similar (cosign similarity ≥0.734) to COSMIC 5. Forty cohort samples were similar (cosign similarity ≥0.796) to COSMIC 6 (Fig. 4E). TCGA AA mutational patterns shared similarities with COSMIC 1 and 5 (cosign similarities ≥0.645), and TCGA EA tumors shared similarities with COSMIC 1, 3, and 4 (cosign similarities ≥0.481; Supplementary Fig. S10). Within the COSMIC database, mutational signatures 1, 5, and 6 were the signatures most often observed in prostate cancer.

To determine the mechanism associated with tumorigenesis in NG prostate cancer tumors, mutated genes present in at least 2 NG patients were analyzed for functional gene ontology enrichment. NG tumors showed significant (*q* < 0.001) GO and functional enrichment in mismatch repair and non-homologous repair deficiency pathways (Fig. 4F; Supplementary Table S10). Additional enriched pathways included PD-1 checkpoint, thyroid hormone signaling, FOXO signaling, ErB2 signaling, adherens junctions, proteoglycans, and sphingolipids.

## Discussion

This study is the first to perform WES of advanced-stage, treatment-naïve primary tumors from NG men with prostate cancer. We analyzed the genomes of 45 tumors and 11 normal NG prostate samples. Because most AAs in the United States have majority West African Ancestry, we assessed genetic admixture on comparison datasets from TCGA, which contains data on AA and EA patients with prostate cancer. We identified ancestry-linked germline and somatic mutation frequencies in DNA damage repair genes (*BRCA1, BRCA2, APC,* and *ATM*), as well as three novel prostate cancer–associated genes (*CACNA2D2, SYNE1,* and *ADAMTS2*). Mutations in DNA damage repair pathways are involved in prostate cancer development and progression and are clinically targetable (64). Because men of African ancestry are severely underrepresented in genomic studies, our findings address a gap in the contribution of genetic variation to the incidence of prostate cancer and aggressiveness of the disease in MAA. Furthermore, these findings encourage us to identify clinically targetable sites to close the gap in health-related disparities.

We observed a high level of *BRCA1* germline mutation in prostate cancer of NG and men of African ancestry. The high rate is driven by the variant BRCA1_rs799917 T>C, which enhances disease risk in triple-negative breast (55), gastric (56), esophageal squamous cell (53), and lung (54) cancers. Results of meta-analyses, however, suggest that this variant is nonpathogenic (65–67). Because none of these studies included patients of African ancestry, the impact of this variant on that population remains poorly explored. BRCA1_rs799917 T>C alters the coding sequence of *BRCA1*, lowering *BRCA1* expression by inhibiting its interaction with miR-638 (68). In addition, because *BRCA1* is a DNA damage repair gene, it has been reported to upregulate the expression of multiple antioxidant genes and oxidoreductases, balancing cellular redox (69). Lower expression of *BRCA1*, skews this balance, which results an increase in DNA-damaging reactive oxygen species (ROS). AA men have been shown to possess lower mtDNA content, which can lead to enhanced ROS production and mitochondrial dysfunction (70). Coupled together, lower *BRCA1* expression and increased ROS production can lead to an accumulation of mutated DNA, which enhances tumorigenesis. BRCA1_rs799917 T>C has not previously been associated with prostate cancer; however, *BRCA1* germline mutations contribute to increased prostate cancer risk (71) and are associated with higher prostate cancer aggression and poorer outcomes (72). We found this trend in AA prostate cancer, which have higher frequencies of germline *BRCA1* VUS (73). This pattern is also evident in both NG and AA breast cancer tumors (74–76). Thus, our exome analysis provides evidence of distinctive germline BRCA1 mutations in prostate cancer of patients with African Ancestry.

Our analysis also showed that, in their DNA damage response (DDR) genes, NG prostate cancer tumors have higher somatic variant rates, with 53% of NG tumors having at least one somatic DDR gene mutation. NG tumors demonstrated increases in *BRCA2, APC,* and *BRCA1* mutations and SNP patterns associated with defective DNA mismatch repair. In addition, these tumors contained mutated genes that had significant gene ontology and functional enrichment across multiple GO groups, including mismatch repair and homologous recombination (HR) signaling pathways. Pathogenic mutations in DDR genes are prevalent in advanced-stage, localized prostate cancer, especially affecting genes responsible for repair by HR (77). Of note, *BRCA2* facilitates the formation of *RAD51* (RAD51 Recombinase) filaments, which are necessary for HR (78). The clinical implications of somatic *BRCA2* mutations are poorly understood; however, all *BRCA2* mutations (germline and somatic) are understood to destabilize HR, increase tumor aggression, and contribute to poor patient outcomes (79). DNA repair signalling is often impaired in cancer cells (80); however, this impairment is more substantial for MAA. Yadav and colleagues observed increased somatic mutations of *BRCA2, BRCA1,* and *ATM* in AA prostate cancer (26). AAs had a 1.24- to 2.16-fold increase in *BRCA2*, *BRCA1*, and *ATM*, as compared with EAs. The innate impairment of DNA repair in cancer cells leads to a dependence on alternative repair pathways that can be therapeutically exploited. Taking our findings, which are in line with other reports for AA men, the recent FDA approval of PARP inhibitors (specifically olaparib) and other therapies such as platinum drugs, ATR inhibitors, CHEK1/2 inhibitors, and radiotherapy may be useful for men of African Ancestry (81, 82).

Our analyses identified, in addition to somatic mutations in DDR genes, somatic mutations in novel prostate cancer–associated genes. Of NG tumors, 27% (*n* = 13) contained a recurrent *CACNA2D2* missense SNP of Leu54Phe (rs569543350). Both ClinVar and dbSNP designate this variant as having “unknown significance.” *CACNA2D2* modulates the expression of functional calcium channels (83), which contribute to cancer development (84). Compared with noncancerous prostate tissue, *CACNA2D2* is expressed higher in prostate cancer tissue and can increase tumor proliferation and angiogenesis (85). *SYNE1* encodes a multi-isomeric protein that participates in connecting the nuclear envelope to the cytoskeleton. This connection is necessary for proper nuclear movement and positioning, and for cellular migration (86). Abnormal nuclear envelope structure, a feature of cancer, is thought to contribute to tumorigenesis (87). Mutations in *SYNE1* are linked to several human cancers (88). *ADAMTS2* encodes a procollagen N-proteinase that is necessary for collagen fibril assembly (89). The role of *ADAMTS2* in cancer remains poorly understood; however, the impact of collagen metabolism is well characterized (90, 91). Collagen is a structural component of the extracellular matrix, and its metabolism can affect tumor development, tumor tissue stiffness, metastasis, and treatment response. The presence of these novel prostate cancer–associated mutations is unsurprising. Non-European populations have higher rates of VUS (92–94). This is a result of the lack of diversity in research and (by extension) genomic databases (95). Non-European populations are underrepresented in research and genomic databases, which skews genomic annotations away from identifying clinically relevant variants in these populations. Further investigation into these novel mutations may expose alternative routes of disease aggression for MAA and mitigate the disparities.

These findings present the most comprehensive characterization of the NG prostate cancer exome to date and highlight the need to increase study population diversity. Although clinical genomics is a powerful tool to guide clinical interventions, the lack of non-European patients limits the capacity of these advancements to benefit men of African ancestry. Furthermore, the high level of genetic diversity within African men necessities the need for larger cohort studies to identify population-specific, recurrent mutations that contribute to prostate carcinogenesis.

Although our results are compelling, the study has some limitations. The high level of genetic diversity in Africans coupled with the lack of matched normal samples increased the number of detected somatic variants, requiring us to aggressively filter variants to limit the possibility of false positives. This conservative approach, although necessary, provides the possibility that we inadvertently excluded some true somatic variants. Moreover, the use of a nonmatched NG normal was employed to further filter out germline variants within somatic calls. These strategies reduced our ability to resolve ethnic and geographical differences within our NG cohort and required that we conduct a supervised analysis of variants within known prostate cancer driver genes. Second, we set a 70% African ancestry threshold for AA samples, (i) to make sure our AA and European TCGA comparison groups were as distinct as possible and (ii) to enrich the AA cohort for African ancestry. Setting this threshold limits the ability to determine the prevalence of the observed variant patterns in more admixed AA samples; however, a review of the available literature shows that the majority of AAs have at least that amount (96, 97). Finally, the limited representation of African patients within genomic databases and genomic research reduces our ability to determine the larger population-specific distribution of these findings. To our knowledge, this is the largest prostate cancer exome study of NG men, and, despite the limitations, provides a robust characterization of the somatic landscape within NG prostate cancer.

## Supplementary Material

Supplementary Tables 1-10 Supplementary Table 1. Known Germline Variants. Supplementary Table 2. Germline Variants. Supplementary Table 3. Known Somatic Variants. Supplementary Table 4. Somatic Vars in NG Normal. Supplementary Table 5. NG WES QC Metrics. Supplementary Table 6. NG Cohort Description. Supplementary Table 7. TCGA Cohort Description. Supplementary Table 8. AA Germ Var Frequency. Supplementary Table 9. Somatic Variants. Supplementary Table 10. NG Somatic ClueGo Results.

Supplementary Figures 1-10 Supplementary Figure 1. WES Analysis Workflow. Supplementary Figure 2. Variant Strand Bias. Supplementary Figure 3. Somatic Variant Filtering. Supplementary Figure 4. Manual Somatic Variant Inspection. Supplementary Figure 5. Genetic Admixture Analysis. Supplementary Figure 6. Comparison of somatic variant count based on normal sample usage. Supplementary Figure 7. TCGA EA Somatic Variant Lollipop Plots. Supplementary Figure 8. NG PCa Novel Somatic Variants. Supplementary Figure 9. Nigerian Germline Variant – Normal/Tumor Comparison Lollipop Plots. Supplementary Figure 10. TCGA PCa Somatic Variant COSMIC Signature Analysis.
