# Supplementary material for: Whole-exome Sequencing of Nigerian Prostate Tumors from the Prostate Cancer Transatlantic Consortium (CaPTC) Reveals DNA Repair Genes Associated with African Ancestry
Source: Cancer Res Commun. 2022 Sep 16;2(9):1005–16. doi: 10.1158/2767-9764.CRC-22-0136 (PMC10010347; doi:10.1158/2767-9764.CRC-22-0136)
Supplement: Supplementary Figures 1-10 — Supplementary Figure 1. WES Analysis Workflow. Supplementary Figure 2. Variant Strand Bias. Supplementary Figure 3. Somatic Variant Filtering. Supplementary Figure 4. Manual Somatic Variant Inspection. Supplementary Figure 5. Genetic Admixture Analysis. Supplementary Figure 6. Comparison of somatic variant count based on normal sample usage. Supplementary Figure 7. TCGA EA Somatic Variant Lollipop Plots. Supplementary Figure 8. NG PCa Novel Somatic Variants. Supplementary Figure 9. Nigerian Germline Variant – Normal/Tumor Comparison Lollipop Plots. Supplementary Figure 10. TCGA PCa Somatic Variant COSMIC Signature Analysis. [file crc-22-0136-s02.docx]

Supplemental Figure 1


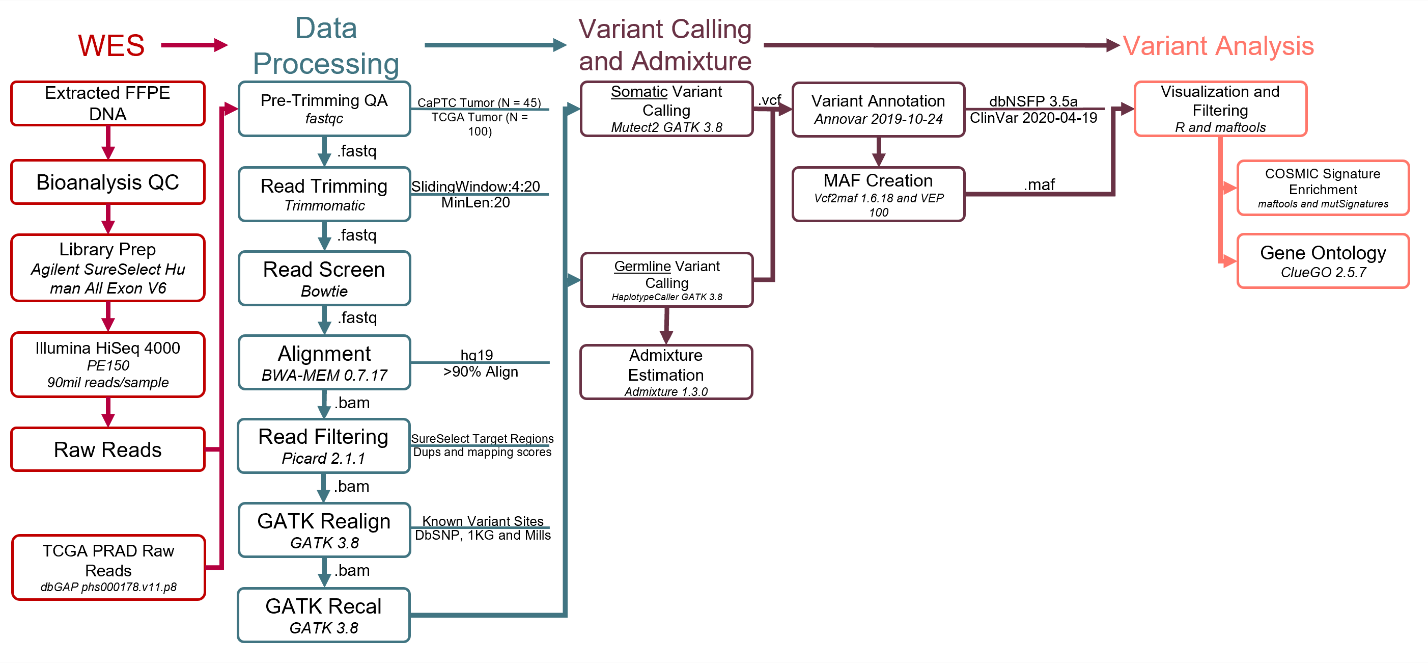


**Supplemental Figure 1 - WES Analysis Workflow.** NG CaPTC and TCGA PCa DNA samples were processed and analyzed using the CCBR WES pipeline in tumor-normal somatic variant and germline modes. Incorporating GATK Best Practices, CCBR expertise, and project specific modifications, these pipelines provided comprehensive raw data quality assurance, variant calling, and genetic admixture estimation. Output mutation tables (maf) and variant calls (vcf) were used for all downstream analyses. CaPTC cohort tumor exomes were paired with a single unmatched normal exome to diminish false-positive somatic variant calls. Patient-matched normals were used for TCGA exomes.

Supplemental Figure 2


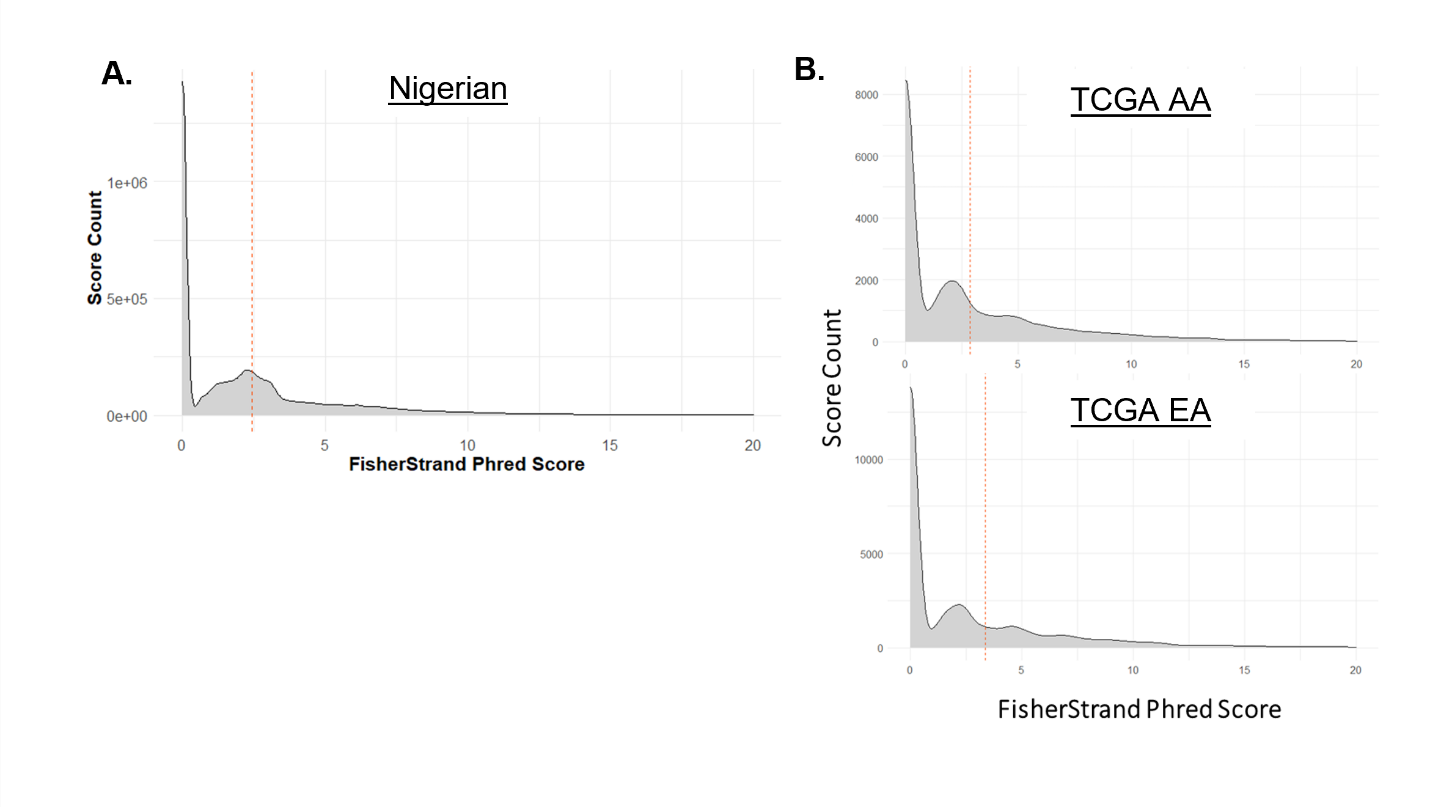


**Supplemental Figure 2 - Variant Strand Bias.** Variant annotation included the FisherStrand metric generated by Mutect2. This phred-scaled Fisher’s Exact Test p-value provides an indicator of strand bias probability. GATK best practices uses an FS score of ≤60 to filter out variants with a high probability of strand bias. **A)** Within the NG cohort, FS scores were well below that threshold, meaning the probability of the reads being false positive is diminished. **B)** FS scores within AA and EA TCGA samples were also below that threshold. The red dashed line denotes the mean FisherStrand Phred score.

Supplemental Figure 3


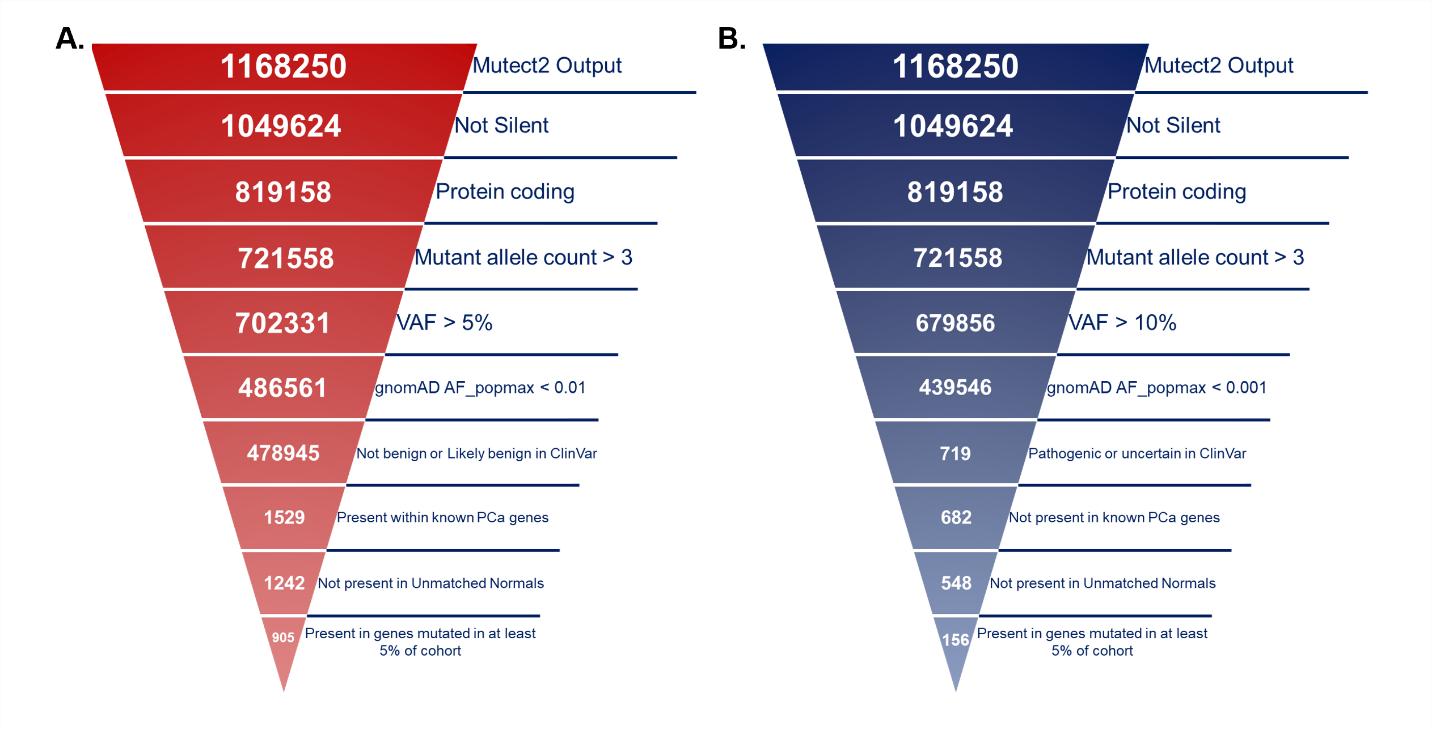


**Supplemental Figure 3 - Somatic** **Variant Filtering**. Two filtering regimes were utilized to identify NG PCa somatic variants within known and novel PCa-associated genes. **A)** To identify variants within known PCa-associated genes, mutated NG cohort genes not identified as harboring pathogenic somatic mutations in ClinVar were excluded, with remaining variants being filtered conservatively. **B)** Variants within novel PCa-associated genes were aggressively filtered and were not in genes identified in ClinVar.

Supplemental Figure 4


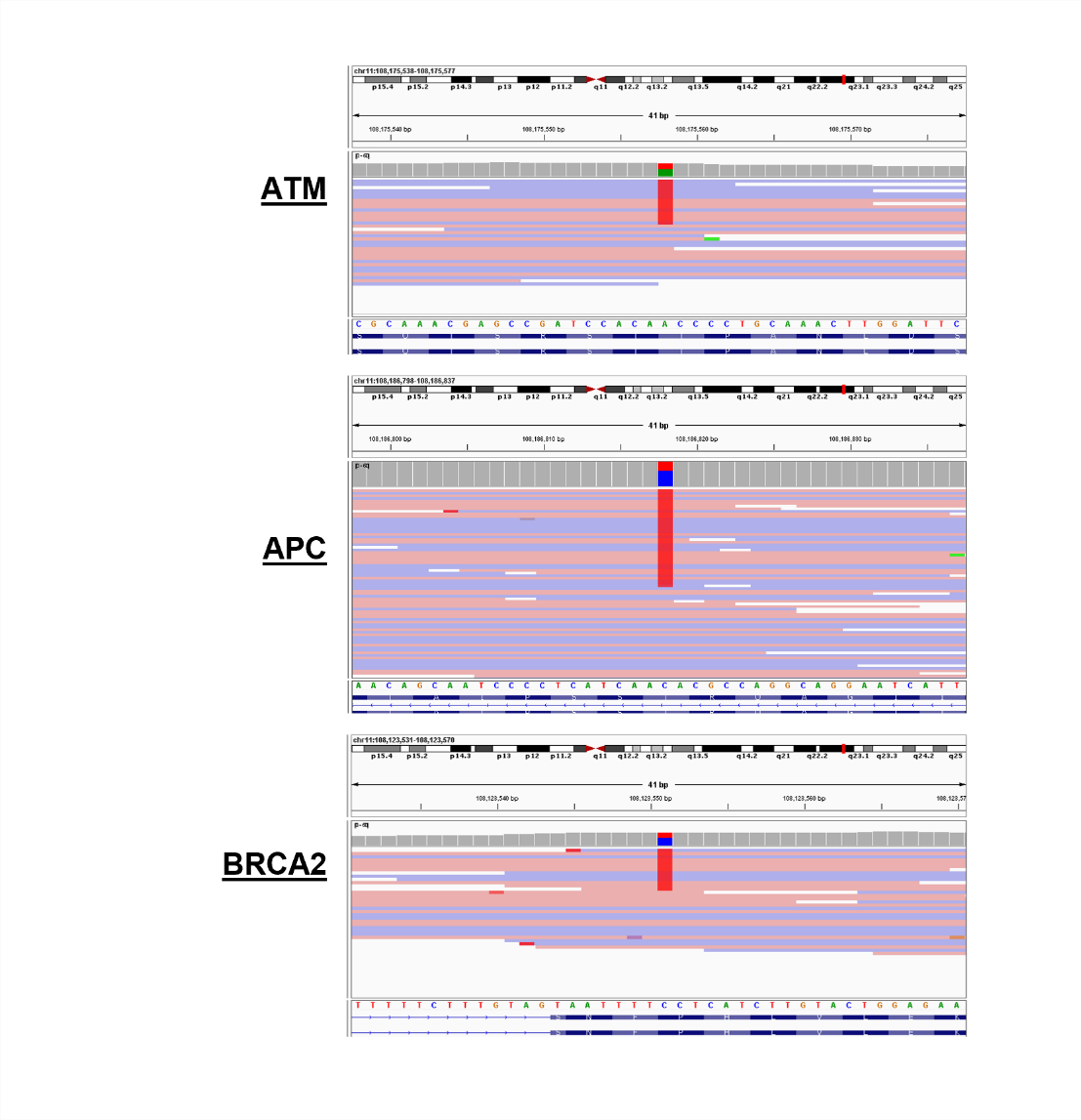


**Supplemental Figure 4 - Manual Somatic Variant Inspection.** Following variant filtration, variants within the most frequently mutated genes were manually inspected in IGV to validate variant calls. If a nucleotide differs from the reference sequence in greater than 20% of quality weighted reads, IGV colors the bar in proportion to the read count of each base (A, C, G, T). A, green; C, blue; G, yellow; T, red.

Supplemental Figure 5


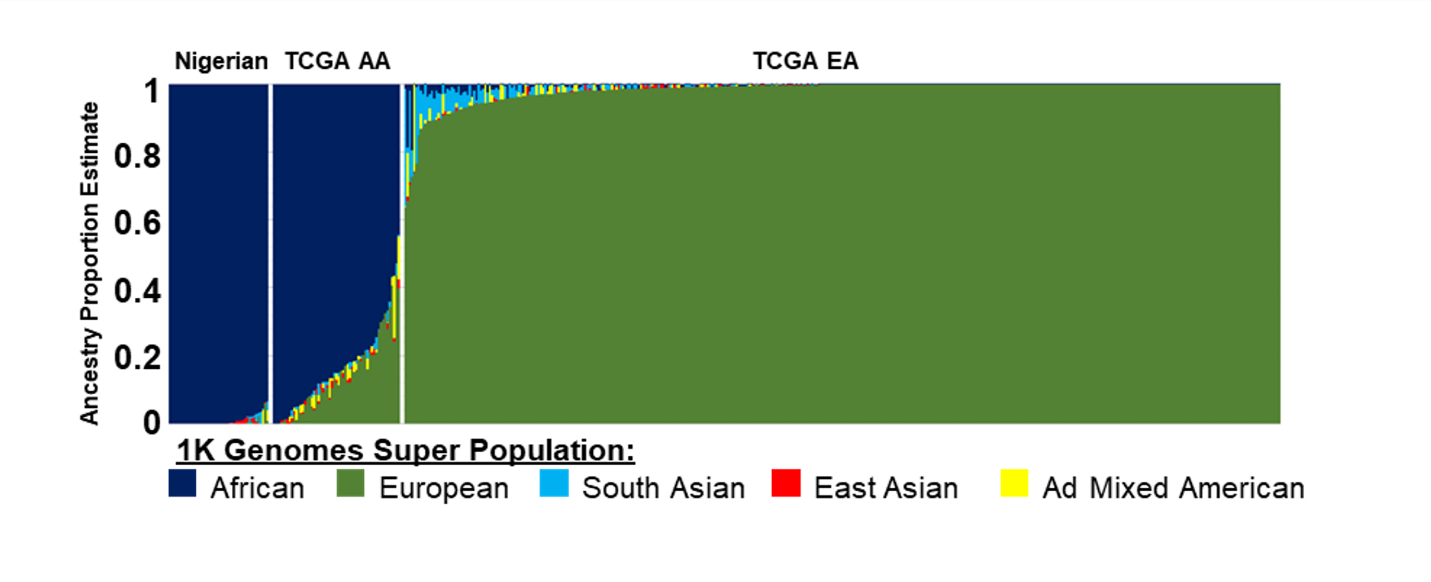


**Supplemental Figure 5 - Genetic Admixture Analysis.** Admixture v1.3.0 was used to estimate ancestry proportions, based on reference populations from the 1000 Genomes Project phase 3 superpopulations. Rare variants (i.e., <5% across all phase 3 1000 genomes), all indels, and any SNPs that were not biallelic, were removed prior to analysis. Samples within the CaPTC cohort had an average African proportion of 99.1%. TCGA Samples (n=50) with >70% African ancestry were classified as AAs. 402 TCGA samples contained >60% European admixture.

Supplemental Figure 6


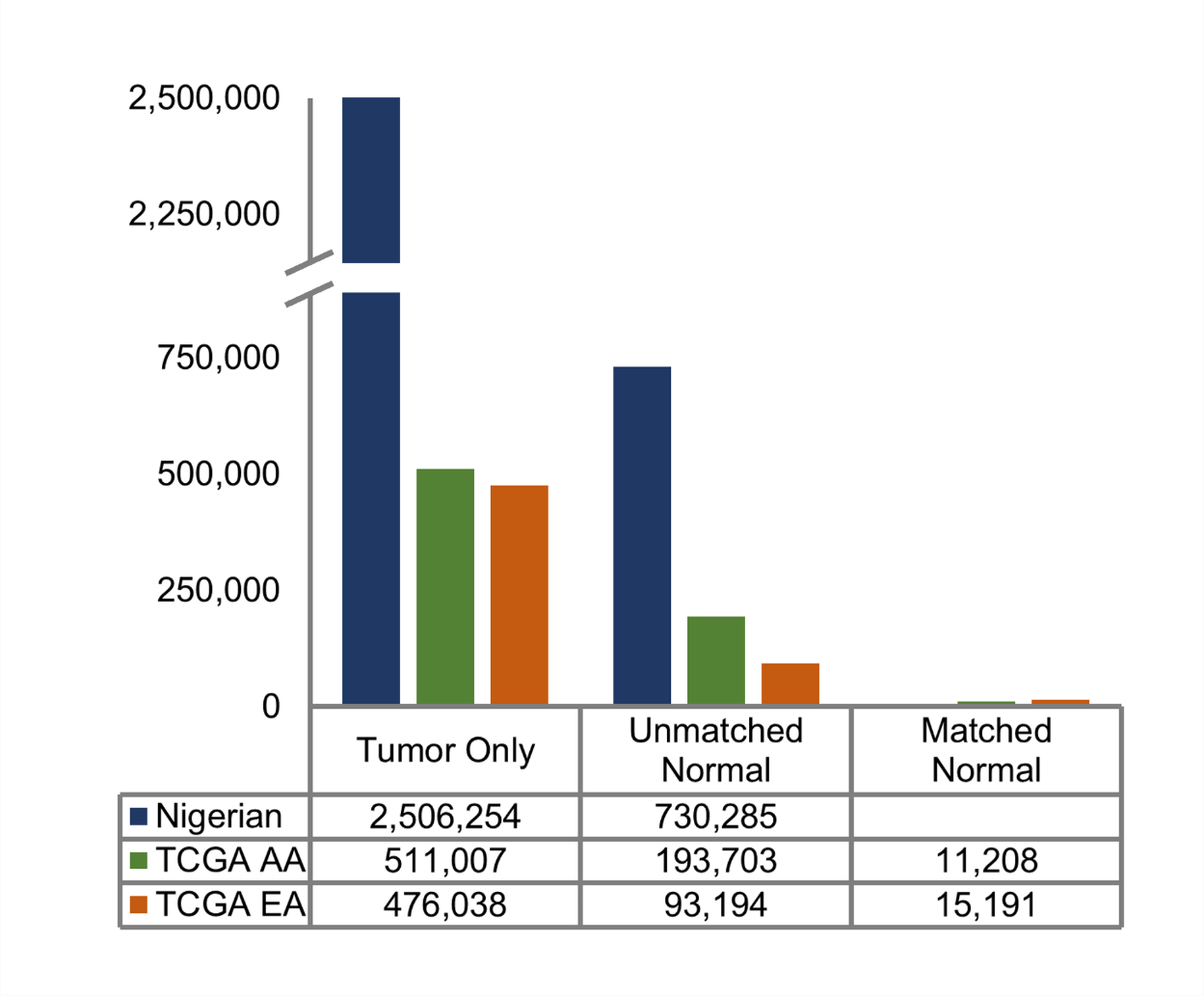


**Supplemental Figure 6 - Comparison of somatic variant count based on normal sample usage.** To determine the impact of normal sample usage on the total number of called variants, each cohort was analyzed up to three times. NG CaPTC samples were analyzed using two methods (1-Tumor-only and 2- Against an unmatched normal). Tumor-only analysis produced 2,506,254 variants; however, that count was reduced by 70.8% when an unmatched normal was used. Each TCGA cohort was analyzed using three methods (1-Tumor only, 2-Against an unmatched normal, and 3-Against a matched normal). For AA TCGA samples, tumor-only analysis produced 511,007 variants; however, that count was reduced by 62.1% and 97.8% with unmatched and matched normals, respectively. For EA TCGA samples, tumor-only analysis produced 476,038 variants; however, that count was reduced by 80.4% and 96.8% with unmatched and matched normals, respectively.

Supplemental Figure 7


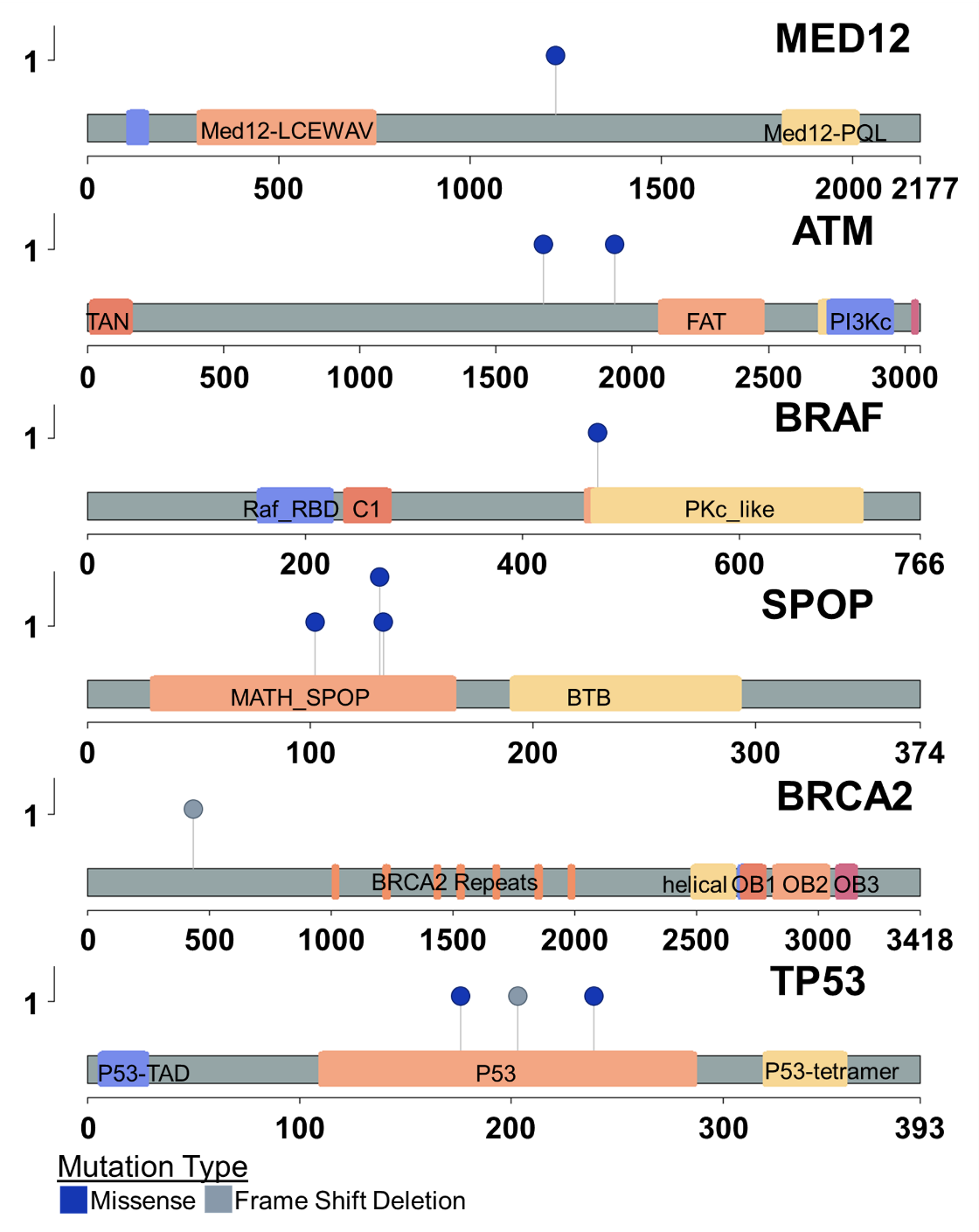


**Supplemental Figure 7 - TCGA EA Somatic Variant Lollipop Plots.** After filtering, the European cohort (n = 50) contained 21,957 variants. Comparison of variants with most frequently mutated NG variants showed no discernable pattern.

Supplemental Figure 8


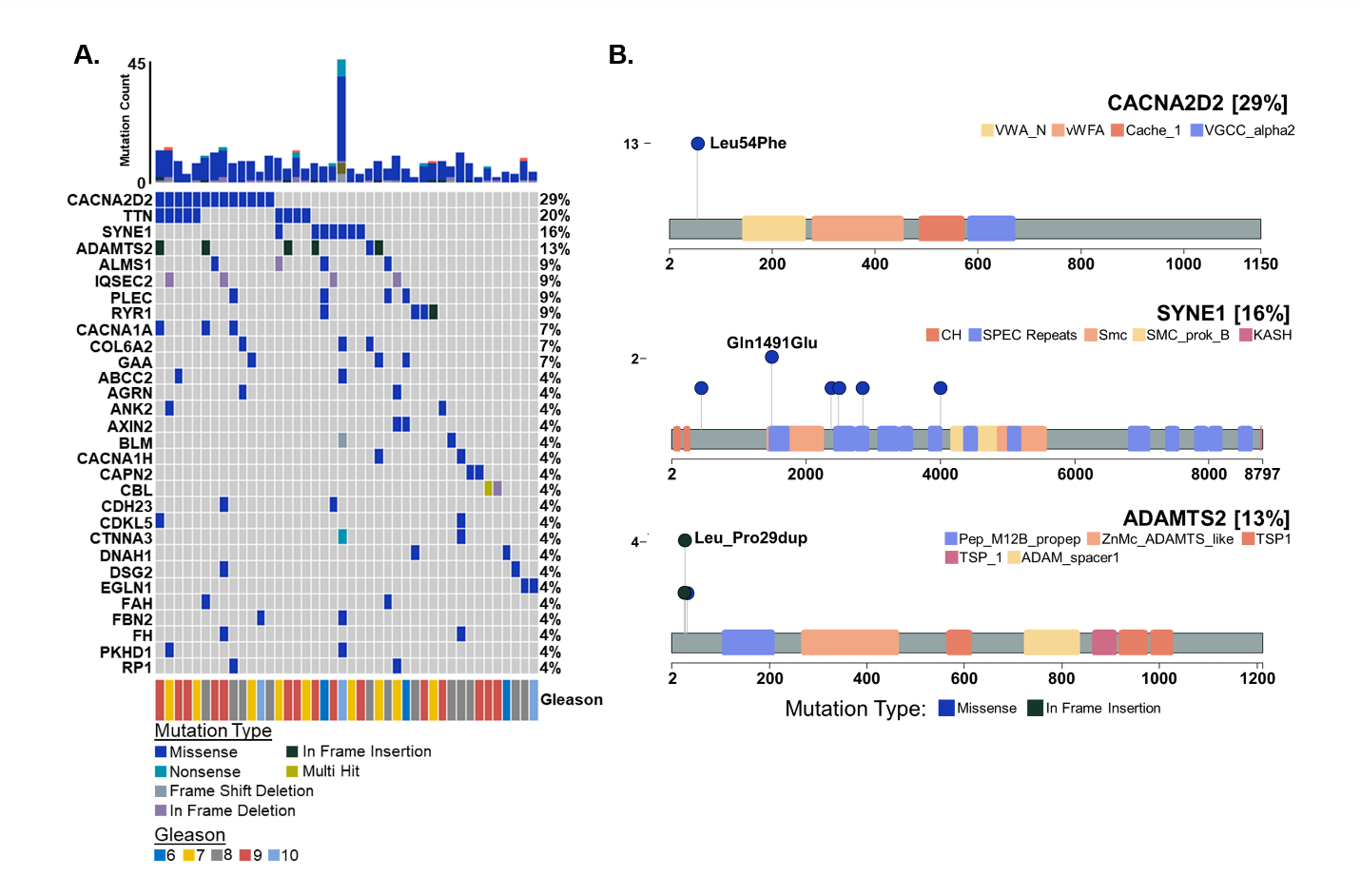


**Supplemental Figure 8 - NG PCa Novel Somatic Variants.** Variant calling within the NG cohort (n=45) produced 1,168,250 variants. NG PCa variants not within canonical genes, identified in ClinVar, were retained and stringently filtered. **A)** 51 genes with novel PCa association harbored variants in at least two tumor samples. **B)** CACNA2D2 (Calcium Voltage-Gated Channel Auxiliary Subunit Alpha2delta 2) had the highest cohort mutation rate of 29%, showing a recurrent (n=13) missense SNP of Leu54Phe. SYNE1 (Spectrin Repeat Containing Nuclear Envelope Protein 1) had a mutation frequency of 16% and a recurrent (n=2) missense SNP of Gln1491Glu. ADAMTS2 (ADAM Metallopeptidase with Thrombospondin Type 1 Motif 2) showed a recurrent (n=4) in-frame insertion of Leu_Pro29dup and an overall mutation frequency of 13%.

Supplemental Figure 9


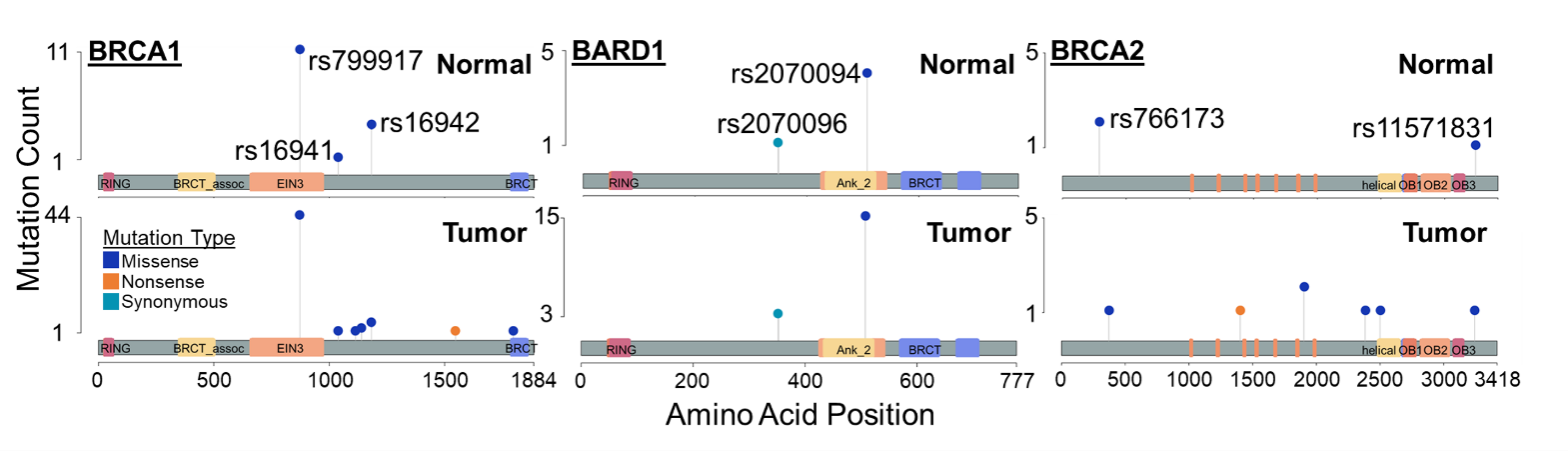


**Supplemental Figure 9 - Nigerian Germline Variant – Normal/Tumor Comparison Lollipop Plots.** To validate germline variants identified within Nigerian (NG) Normal (n=11) samples, NG tumor (n=45) samples were processed using the same pipeline and surveyed for variant frequencies in *BRCA1, BARD1,* and *BRCA2*. NG tumor samples, like NG normal samples, showed a high frequency (100%) of rs799917, moderate frequencies (30-36%) of rs2070094 and rs16942, and low frequencies (9-2%) of rs16941, rs2070096, and rs11571831. rs766173 was not present in NG tumor samples.

Supplemental Figure 10


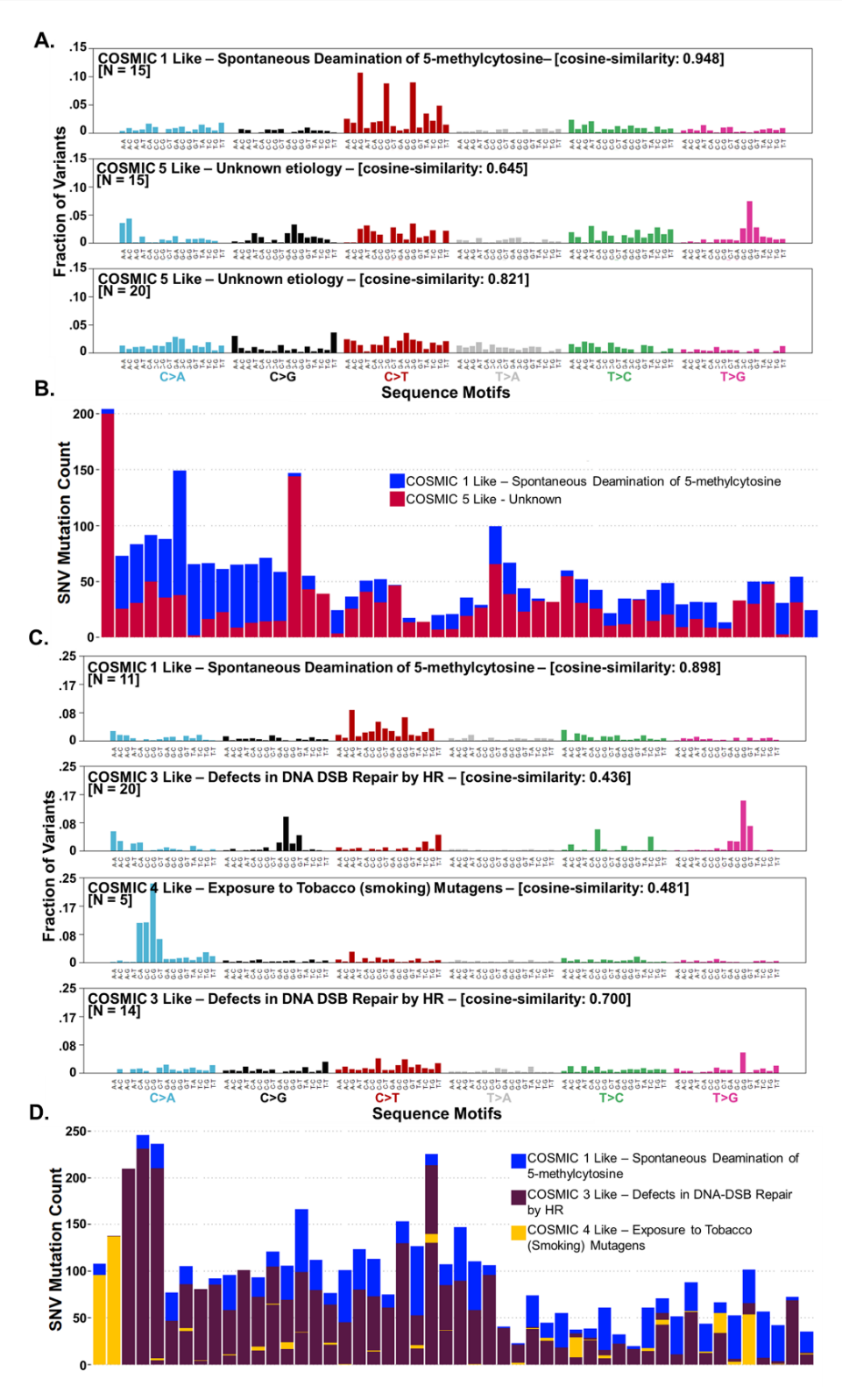


**Supplemental Figure 10 - TCGA PCa Somatic Variant COSMIC Signature Analysis**. Single nucleotide variations in both TCGA PCa cohorts were compared to known cancer-related mutation signatures within the Catalogue of Somatic Mutations in Cancer (COSMIC). **A and B)** TCGA AA PCa mutation patterns shared similarities (cosign similarities ≥ 0.645) with COSMIC signatures 1 and 5. **C and D)** TCGA EA PCa mutation patterns shared similarities (cosign similarities ≥ 0.436) with COSMIC signatures 1, 3, and 4.
